# Relief for the Dentist

**Published:** 1878-10

**Authors:** 


### RELIEF FOR THE DENTIST.

Everything that tends to lighten the labor of the dentist should be received, with just appreciation of the efforts of him who originates and constructs.

In a recent number of the REGISTER we made some reference to the dental operating chair, its desirable qualities, etc. Since that time Dr. S. W. How, of this city, has attached to our chair—an "S. S. White"—three accessories, in the form of spiral springs, that render the movements more easily made. One of these consists of a strong spiral spring of heavy steel wire, which rests on the base and surrounds the support shaft, and exerts a strong lifting force against the body of the chair, thus its elevation is very easy even with an ordinary person on the seat; the upward and downward movement is made with about the same ease. Another spiral spring is placed in the socket that receives the rod that supports the head-rest; this brings the head-rest to a self-balance at about medium height; and a very slight effort moves it up or down to its extremes.

Another spring is attached to the rear of the chair at the left side which raises the back and balances it at a medium of its working distance; by this arrangement it is also raised or lowered to any point in its working range, by the application of a few ounces of force, whereas without this appliance a lift of from ten to fifteen pounds is necessary to raise the back. In these three particulars, the parts being placed on springs at a medium working point, it is a very easy matter to move them to either extreme, and thus great relief is afforded in working the chair. This is a matter of more importance than is usually attached to it.

In order to have the best control of his manipulative ability during operation in the mouth, the dentist should be as free as possible from extra muscular efforts, and in the use of these appliances of Dr. How, much relief in this direction is given.

### THE MISSOURI DENTAL JOURNAL.

This journal has passed into the hands of Prof. C. W. Spalding as editor and proprietor. With this change we think the profession will find no fault.

The *Missouri Journal* has been an agent of great benefit to the profession of the West. It has always contained much interesting and instructive matter.

We feel assured that it will not retrograde in the present hands.

Dr. Spalding is well and extensively known throughout the profession of this country, and there will be a general anxiety to see whatever emanates from his pen.

We trust that the *Missouri Journal* and the DENTAL REGISTER will be intimate friends. *So mote it be.*

